# Avenanthramides Prevent Osteoblast and Osteocyte Apoptosis and Induce Osteoclast Apoptosis in Vitro in an Nrf2-Independent Manner

**DOI:** 10.3390/nu8070423

**Published:** 2016-07-11

**Authors:** Gretel G. Pellegrini, Cynthya C. Morales, Taylor C. Wallace, Lilian I. Plotkin, Teresita Bellido

**Affiliations:** 1 Department of Anatomy & Cell Biology, School of Medicine, Indiana University, Indianapolis, IN 46202, USA; moralecy@iupui.edu (C.C.M.); lplotkin@iupui.edu (L.I.P.); 2 Roudebush Veterans Administration Medical Center, Indianapolis, IN 46202, USA; 3 Department of Nutrition and Food Studies, George Mason University, Fairfax, VA 22030, USA; taylor.wallace@nof.org; 4 Think Healthy Group, LLC, Washington, DC 20001, USA; 5 National Osteoporosis Foundation, Arlington, VA 22202, USA; 6 Department of Medicine, Division of Endocrinology, School of Medicine, Indiana University, Indianapolis, IN 46202, USA

**Keywords:** avenanthramides, oxidative stress, apoptosis, gene expression, bone cells

## Abstract

Oats contain unique bioactive compounds known as avenanthramides (AVAs) with antioxidant properties. AVAs might enhance the endogenous antioxidant cellular response by activation of the transcription factor Nrf2. Accumulation of reactive oxygen species plays a critical role in many chronic and degenerative diseases, including osteoporosis. In this disease, there is an imbalance between bone formation by osteoblasts and bone resorption by osteoclasts, which is accompanied by increased osteoblast/osteocyte apoptosis and decreased osteoclast apoptosis. We investigated the ability of the synthethic AVAs 2c, 2f and 2p, to 1-regulate gene expression in bone cells, 2-affect the viability of osteoblasts, osteocytes and osteoclasts, and the generation of osteoclasts from their precursors, and 3-examine the potential involvement of the transcription factor Nrf2 in these actions. All doses of AVA 2c and 1 and 5 µM dose of 2p up-regulated collagen 1A expression. Lower doses of AVAs up-regulated OPG (osteoprotegerin) in OB-6 osteoblastic cells, whereas 100 μM dose of 2f and all concentrations of 2c down-regulated RANKL gene expression in MLO-Y4 osteocytic cells. AVAs did not affect apoptosis of OB-6 osteoblastic cells or MLO-Y4 osteocytic cells; however, they prevented apoptosis induced by the DNA topoisomerase inhibitor etoposide, the glucocorticoid dexamethasone, and hydrogen peroxide. AVAs prevented apoptosis of both wild type (WT) and Nrf2 Knockout (KO) osteoblasts, demonstrating that AVAs-induced survival does not require Nrf2 expression. Further, KO osteoclast precursors produced more mature osteoclasts than WT; and KO cultures exhibited less apoptotic osteoclasts than WT cultures. Although AVAs did not affect WT osteoclasts, AVA 2p reversed the low apoptosis of KO osteoclasts. These in vitro results demonstrate that AVAs regulate, in part, the function of osteoblasts and osteocytes and prevent osteoblast/osteocyte apoptosis and increase osteoclast apoptosis; further, these regulatory actions are independent of Nrf2.

## 1. Introduction

Oxidative stress is caused by the imbalance between free radical generation and the scavenging activities of intracellular antioxidants and plays a critical role in many chronic and degenerative diseases, including osteoporosis, cancer, and neurodegenerative diseases [1,2,3]. High levels of reactive oxygen species (ROS), especially hydrogen peroxide (H_2_O_2_), in the bone/bone marrow microenvironment play a pathogenic role in osteoporosis due to estrogen deficiency with menopause, androgen deficiency during aging in both women and men [4], and/or glucocorticoid therapy used in treating many inflammatory and autoimmune diseases [5]. ROS accumulation increases the number of osteoclasts (the cells that resorb bone) by enhancing the expression by osteoblastic cells of the pro-osteoclastogenic cytokines RANKL (Receptor Activator for Nuclear Factor κB Ligand) and TNFα (Tumor Necrosis Factor alpha) [6,7,8]. ROS increase osteoclast differentiation directly by activating the transcription factor NFATc1, which in turn increases transcription of osteoclast-specific genes [6]. ROS also promotes osteoclast survival. On the other hand, ROS accumulation decreases the number of osteoblasts (the cells that form bone) by inhibiting their proliferation and differentiation; and induces premature osteoblast apoptosis [9,10,11,12]. In addition, ROS induces apoptosis of osteocytes, the most abundant cells in the bone that orchestrate osteoclast and osteoblast function [13,14]. 

Two main therapeutic approaches have been developed for the management of osteoporosis: (1) Anti-resorptive medications including bisphosphonates, estrogen replacement, and anti-RANKL antibodies; and (2) Anabolic treatments such as daily injections of parathyroid hormone (PTH) [15]. The first approach seeks to block osteoclast formation and/or function and the second stimulates osteoblast production and function. Although therapies for treating osteoporosis have been shown to be effective, prevention strategies through optimal lifestyle patterns (e.g., nutrition and physical activity) are actively being sought to help decrease the overall burden of osteoporosis and high bone fracture risk. There is an increase in the prevalence of degenerative diseases that affect bone and involve increased oxidative stress. Therefore, alternative therapeutic interventions that counteract ROS effects in bone without causing harmful effects in other tissues are needed.

Nuclear factor erythroid derived 2-related factor-2 (Nrf2) plays an important role in the cellular defense against oxidative stress by inducing enzymes that regulate oxidative stress [16,17]. Recent studies have demonstrated that Nrf2 is an important regulator of bone homeostasis in bone cells, since activation of Nrf2 can enhance the endogenous antioxidant response against ROS [1,16,18,19].

Increasing evidence, including epidemiological, clinical, and animal experimentation, suggests that consumption of plant foods, containing polyphenols helps to protect against the development of oxidative stress pathologies, including cancer, cardiovascular diseases, diabetes, neurodegenerative diseases, and osteoporosis [20,21,22,23]. Oat is a commonly consumed whole-grain cereal that is gaining scientific and public interest for their health benefits beyond basic nutrition [24,25]. Oats contain phytochemicals with high antioxidant properties, among them tocopherols, tocotrienols, phenolic compounds, phytic acid, avenanthramides (AVAs), and flavonoids and sterols in a lesser amount [24,26,27]. AVAs are a group of alkaloid phenols uniquely found in oats [28]. These compounds consist of an anthranilic acid derivate and a hydroxycinnamic acid derivate linked by a pseudo-peptide bond [29], and exhibit strong antioxidant activity and anti-inflammatory and anti-proliferative properties both in vitro and in vivo [25,30,31,32]. AVAs inhibit the expression of adhesion molecules and inflammatory cytokines, such as IL-6, IL-8, and monocyte chemoattractant protein 1 in human aortic endothelial cell cultures [33]; and inhibit the growth of human colon cancer cells in vitro [28]. Further, dietary intake of AVAs by postmenopausal women decreased the inflammatory response induced by physical exercise and increased the total antioxidant capacity of plasma and the superoxide dismutase (SOD) activity of red blood cells [34]. Addition of AVAs extracts to mouse diets enhanced the hepatic mRNA expression of Cu-Zn SOD1, Mn SOD2, and glutathione peroxidase (GPx) [35]. AVAs also increase hemeoxygenase (HO) 1 expression in human kidney cells in a dose- and time-dependent manner and induce the nuclear translocation of the transcription factor Nrf2 [36].

To date, there are no studies addressing the effect of AVAs on bone cells in vitro. The aims of this study, were to investigate the ability of the three major synthethic AVAs (2c, 2f and 2p), which differ in the type of hydroxylcinnamic acid component (ferulic, caffeic or p-coumaric acid), to 1-regulate gene expression in bone cells, 2-affect the viability of osteoblasts, osteocytes and osteoclasts, and the generation of osteoclasts from their precursors, and 3-examine the potential involvement of the transcription factor Nrf2 in these actions.

## 2. Material and Methods

### 2.1. AVAs Preparation and Cell Treatment

The synthethic AVA 2f, 2c and 2p were provided by Quaker Oats Center of Excellence (Barrington, IL, USA). Purity was controlled by HPLC measurements (2f > 95.1%; 2c > 94.9%; 2p > 96%). AVAs were dissolved in dimethylsulfoxide (DMSO) and added to the cell culture medium with a maximum final DMSO concentration of 0.1%, which showed no cytotoxicity [37]. AVAs were kept as 0.05 M stock solutions and stored at −80 °C until used. Osteoblastic and osteocytic cells were treated with doses ranging from 1 to 100 µM of the AVAs. The dose range was chosen based on previous studies using AVAs in other cell systems [32,36].

### 2.2. Cell Culture 

Adherent primary osteoblastic C57BL/6 wild type (WT) and Nrf2 knock-out (KO) cells were seeded at a density of 1.5 × 10^4^/cm^2^ before being prepared as previously described in the scientific literature [38,39,40]. The cells were cultured in growth medium consisting of α-Minimum Essential Medium Eagle (α-MEM) supplemented with 10% fetal bovine serum (FBS), (Invitrogen, Carlsbad, CA, USA), 1% penicillin/streptomycin (Invitrogen) and 50 mg/mL normocin (Invivogen, San Diego, CA, USA). 

Adherent OB-6 osteoblastic cells were seeded at a density of 5000 cells/cm^2^ in α-MEM supplemented with 10% FBS and MLO-Y4 cells were seeded at a density of 1 × 10^4^ to 2 × 10^4^ cells/cm^2^ on collagen type I—coated plates in α-MEM supplemented with 2.5% FBS and 2.5% bovine calf serum (BCS, Invitrogen), as previously published [41,42,43]. 

WT or KO bone marrow precursors were seeded at a density of 4 × 10^5^/cm^2^ and cultured for 48 h in α-MEM supplemented with 15% FBS, and 1% penicillin/streptomycin and 50 mg/mL normocin. Next, “non adherent cells”, were collected and seeded at a density of 6 × 10^5^/cm^2^ and cultured with α-MEM with 10% FBS and 1% penicillin/streptomycin, and 80 ng/mL of recombinant murine soluble Receptor Activator for Nuclear Factor κB Ligand (sRANKL) (PreproTech, Rocky Hill, NJ, USA) and 20 ng/mL recombinant murine Macrofage Colony Stimulating Factor (M-CSF) (PreproTech). Medium was changed every 2 days for 5 days, as previously reported [44].

### 2.3. RNA Extraction and Quantitative RT-PCR (qPCR) 

To determine the effects of AVAs on gene expression, total RNA was purified from cell preparations using Trizol reagent (Invitrogen) according to the manufacturer’s instructions. RNA was reverse-transcribed using a High Capacity cDNA Archive Kit (Applied Biosystems, Foster City, CA, USA). Gene expression was analyzed by quantitative PCR using the ΔCt method as previously described with Mrps2 (mitochondrial ribosomal protein S2) or GAPDH (Glyceraldehyde-3-phosphate dehydrogenase) as housekeeping genes [45,46]. The following primer probe sets were purchased from Applied Biosystems: Collagen 1A (COL1A) (Mm00801666 g1); Osteocalcin (OCN) (AIT97T5); Osteoprotegerin (OPG) (Mm01205928 m1); RANKL (Mm00441906 m1); Cathepsin K (Cat K) (Mm00484036 m1); Tartrate-resistant acid phosphate (TRAPase) (Mm00475698 m1); Nrf2 (Mm00477784 m1); Mps2 (Mm00475529 m1); GAPDH (Mm99999915 g1). For the other genes, primer probe sets were designed using the Assay Design Center of Roche Applied Science (Indianapolis, IN, USA), and were as follows. For OSTERIX: probe 106, forward primer: gggaagggtgggtagtcatt, reverse primer: ctcctgcaggcagtcctc; Runt-related transcription factor 2 (RUNX2): probe 34, forward primer: tgcctggctcttcttactgag, reverse primer: gcccaggcgtatttcaga; Calcitonin receptor (CAL R): probe 15, forward primer: agaactggagttgggctcac, reverse primer: ggttccttctcgtgaacaggt; NAD(P)H dehydrogenase, quinone 1 (NQO1): probe 50, forward primer: agtacaatcagggctcttctcg, reverse primer: agcgttcggtattacgatcc; HO-1: probe 17, forward primer: tgtgttcctctgtcagcatca, reverse primer: aggctaagaccgccttcct; SOD1: probe 49, forward primer: tgcccaggtctccaacat, reverse primer: caggacctcattttaatcctcac. Glutathione S-transferase 1 (GSTP1): probe 105, forward primer: ggacagcagggtctcaaaag, reverse primer: tgtcaccctcatctacaccaac.

### 2.4. TRAPase Staining and Osteoclast Counting

After 24 h-treatment with AVAs 2f, 2c or 2p at 1 µM or vehicle (DMSO), mature osteoclasts differentiated from WT or KO bone marrow precursors, were examined for the activity of the osteoclast-specific enzyme TRAPase [47]. Briefly, cells were washed with PBS and fixed in 10% formaldehyde for 10 min. Next, cells were stained using a commercial TRAPase staining kit (Sigma-Aldrich, St. Louis, MO, USA) and counterstained with hematoxylin according to the manufacturer’s instructions. TRAPase + cells were considered osteoclasts if having more than 3 nuclei. Osteoclast apoptosis was assessed by quantifying the percentage of osteoclasts that presented at least 50% condensed chromatin and dark nuclei. 

Results are expressed as number of osteoclast/well or as percentage of apoptotic osteoclasts. Images were acquired using a Zeiss Axiovert 35 microscope equipped with a digital camera. 

### 2.5. Quantification of Osteoblastic Cell Viability 

Osteoblastic cells (OB-6 and WT and KO primary cells) and osteocytic cells were seeded in a 48-well plate at a density of 1 × 10^4^ cells/well in αMEM + PSG + 10% FBS and cultured for 20 h. Next, the medium was changed to αMEM + PSG + 2% FBS + each AVA 2c, 2f, 2p at 1, 10, or 100 μM or the vehicle (DMSO) for 1 h. Cells were then exposed to the pro-apoptotic agents dexamethasone (10^−6^ M), etoposide (50 μM), or H_2_O_2_, (50 μM) or vehicle (DMSO) for an additional 6 h. Non-adherent cells were combined with adherent cells released from the culture dish using trypsin—Ethylenediaminetetraacetic acid (EDTA), re-suspended in medium containing serum, and collected by centrifugation. Subsequently, 0.04% trypan blue was added and the percentage of cells exhibiting both nuclear and cytoplasmic staining was determined using a hemocytometer. Cells that excluded the dye were considered alive, and cells stained were considered dead. Data is reported as the percentage of dead cells [48].

### 2.6. Statistical Analysis

Statistical analysis was performed using SigmaPlot (Systat Software Inc., Version 12.0, San Jose, CA, USA). All the results are presented as mean ± standard deviation of multiple cultures. At least 3 experiments per cell type with 5 independent replicates per experiment were performed for gene expression and apoptosis of osteoblastic and osteocytic cells. Three independent replicates were performed for osteoclast quantification and apoptosis. Sample differences were determined by one or two-way ANOVA, followed by pairwise multiple comparisons using Holm-Sidak or Tukey method, depending on the number of variables. Means were considered significantly different at *p* < 0.05. 

## 3. Results

### 3.1. AVAs Regulate OPG and RANKL Gene Expression in OB-6 Osteoblastic and MLO-Y4 Osteocytic Cells

**Figure 1:**
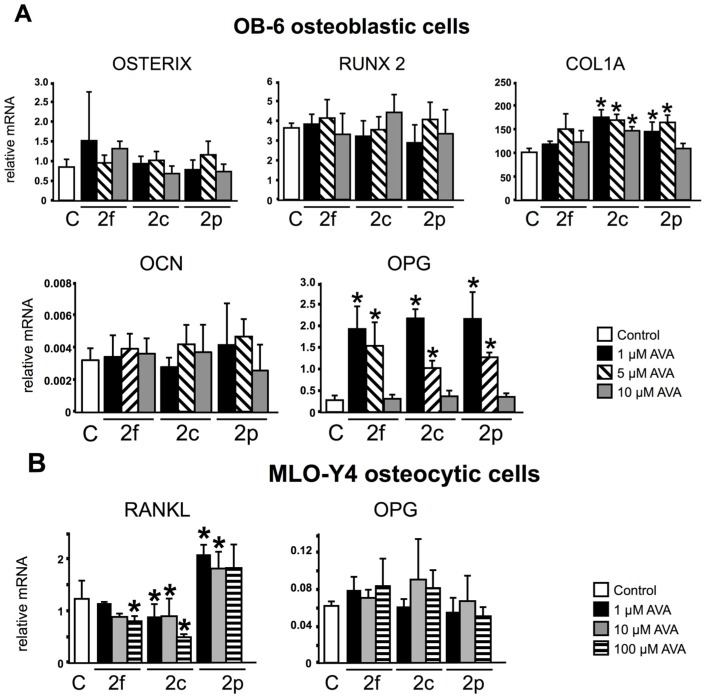
AVAs (Avenanthramides) regulate Collagen 1A (COL1A), osteoprotegerin (OPG) and Receptor Activator for Nuclear Factor κB Ligand (RANKL) in osteoblastic and osteocytic cells, respectively. 24-h gene expression of OB-6 osteoblastic (**A**), and MLO-Y4 cells (**B**); treated with vehicle (**C**) or AVA 2f, 2c or 2p. mRNA levels were measured by qPCR and corrected by Mrps2. The bars represent means ± SD, *n* = 5 replicates/treatment. * *p* < 0.05 vs. control, by One-Way ANOVA, followed by pairwise multiple comparisons using Holm-Sidak method.

Gene expression analysis in OB-6 cells did not show significant changes on the expression of the osteoblast markers OCN, RUNX2 or osterix (Figure 1A), whereas RANKL was not detected in these cells (not shown). On the other hand, AVA 2c and 2p (1, 5 and 10 µM and 1 and 5 µM, respectively) increased COL1A expression. Further, lower doses of the three compounds upregulated OPG expression with more potency than higher doses in OB-6 cells, showing an inverse dose-effect relationship. The reason behind this unexpected biological response is not known and could be due to the involvement of two different mechanisms or molecular mediators, one operating at lower doses and another at higher doses of the compounds. In MLO-Y4 cells, AVAs 2f (100 µM) and 2c (at all concentrations) downregulated the expression of RANKL, whereas AVA 2p (at 1 µM) increased it (Figure 1B). No statistical differences were found for OPG in MLO-Y4 cells. These results suggest that AVAs regulate in part the function of osteoblasts and osteocytes.

### 3.2. AVAs Do Not Affect Cell Death in the Absence of Pro-Apoptotic Agents but Prevent the Effect Induced by Pro-Apoptotic Agents in Ob-6 Osteoblastic and Mlo-Y4 Osteocytic Cells

**Figure 2:**
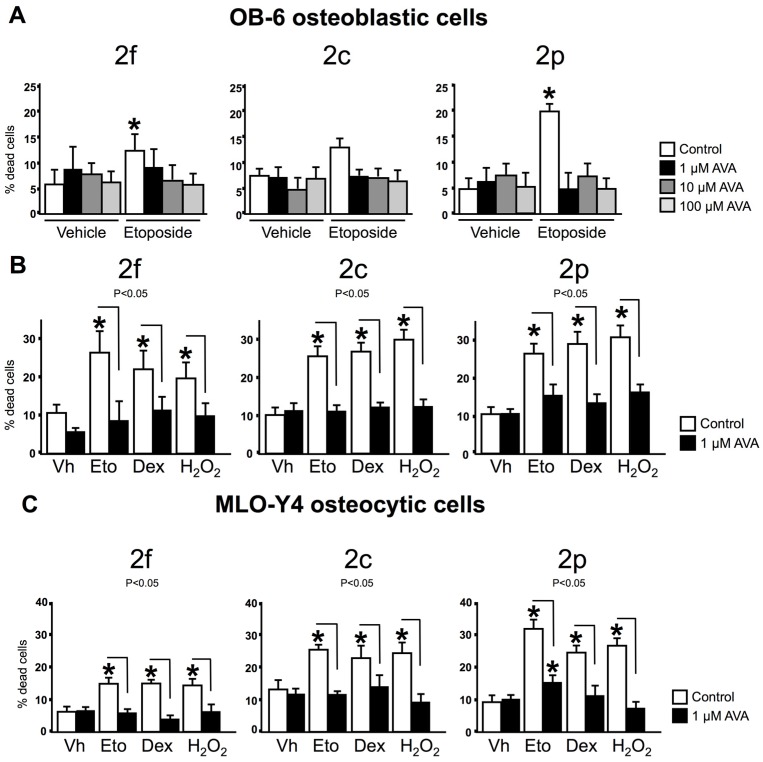
AVAs do not induce apoptosis and prevent cell death induced by proapototic agents in osteoblastic and osteocytic cells. (**A**) Cell death was examined in osteoblastic cells pretreated for 1-h with vehicle (control) or 3 different doses of AVA 2f, 2c and 2p, followed by exposure to vehicle (Vh, dimethylsulfoxide (DMSO)) or etoposide for 6-h; (**B**,**C**) Cells were treated with vehicle (control) or 1 μM AVAs for 1 h, followed by 6 h-treatment with the indicated compounds. Vh, vehicle; Eto, etoposide; Dex, dexamethasone or H_2_O_2_. Cell death was assessed by trypan blue uptake. Bars represent the means ± SD of *n* = 6 independent wells/treatment. * *p* < 0.05 vs. Vh control, and lines connect conditions with significant differences by One-Way ANOVA, followed by Tukey method.

AVAs were further investigated for their effects on osteoblast and osteocyte survival in the absence or in the presence of pro-apoptotic agents. One h pre-treatment with AVA 2f, 2c or 2p at the concentrations tested (1, 10 and 100 µM) did not affect the survival of osteoblastic cells in the absence of pro-apoptotic agents (Figure 2A). As previously reported, the pro-apoptotic agent etoposide, increases the percentage of cells with increased membrane permeability [49]. However, the three AVA compounds, at the same doses, prevented etoposide induced-apoptosis. Since the lowest concentration of AVAs (1 µM) effectively blocked apoptosis of osteblastic cells, this dose was used for the next set of experiments, aiming to examine whether AVAs regulate survival in the presence of the pro-apoptotic agents dexamethasone or H_2_O_2_. Six h-treatment with dexamethasone or H_2_O_2_ increased significantly the percentage of cells exhibiting trypan blue uptake; however 1-h pre-treatment with AVAs prevented dexamethasone or H_2_O_2_-induced OB-6 and MLO-Y4 cell death (Figure 2B,C). These findings demonstrate that AVAs 2f, 2c and 2p preserve the viability of osteoblastic and osteocytic cells in vitro.

### 3.3. The Survival Effect of Avas in Osteoblastic Cells Does Not Require Nrf2 Expression

**Figure 3:**
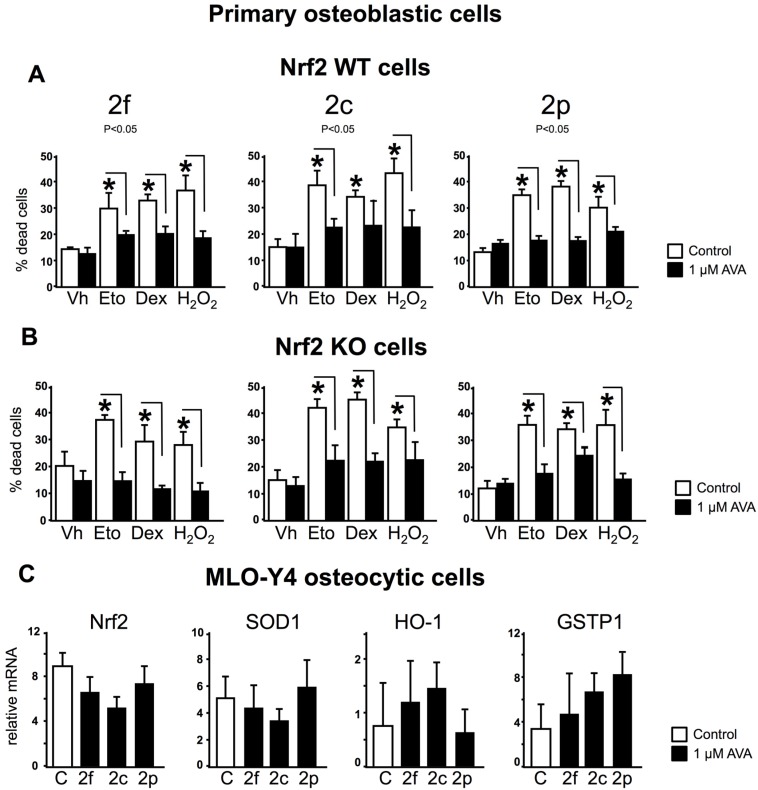
The survival effect of AVAs in osteoblastic cells does not require the expression of Nrf2. (**A**,**B**) Cell death was quantify by trypan blue uptake. Bars represent means ± SD of *n* = 6 samples per treatment. * *p* < 0.05 vs. cells treated with vehicle, by One-Way ANOVA, followed by Tukey method; (**C**) MLO-Y4 osteocytic cells mRNA levels were measured by qPCR and corrected by Mrps2. The bars represent means ± SD, *n* = 5 replicates/treatment. * *p* < 0.05 vs. control, and lines connect conditions with significant differences by One-Way ANOVA, followed by pairwise multiple comparisons using Holm-Sidak method.

We examined whether activation of the Nrf2 pathway, a key component of the antioxidant cellular defense mechanism, was involved in the protective effects of AVAs on osteoblastic cells, by comparing the effects of AVAs in WT or Nrf2 KO osteoblastic cells. Pre-treatment with AVA 2f, 2c or 2p prevented cell death induced by etoposide, dexamethasone or H_2_O_2_ in WT primary osteoblastic cells (Figure 3A). Surprisingly, AVAs were also effective in promoting survival in primary KO osteoblastic cells (Figure 3B). Moreover, AVA 2f was more effective to decrease cell death in the absence or in the presence of pro-apoptotic agents, in primary KO osteoblastic cells compared to WT cells. These findings demonstrate that the survival effect of AVAs on osteoblastic cells does not require Nrf2 expression. Consistent with this conclusion, the mRNA expression of the Nrf2 target genes, cytoprotective enzymes SOD1, HO-1 or GSTP1 were not affected by treatment with AVAs in MLO-Y4 osteocytic cells (Figure 3C). 

### 3.4. Nrf2 Is Not Required for the Regulation of Gene Expression and Survival of Osteoclasts by AVAs

**Figure 4:**
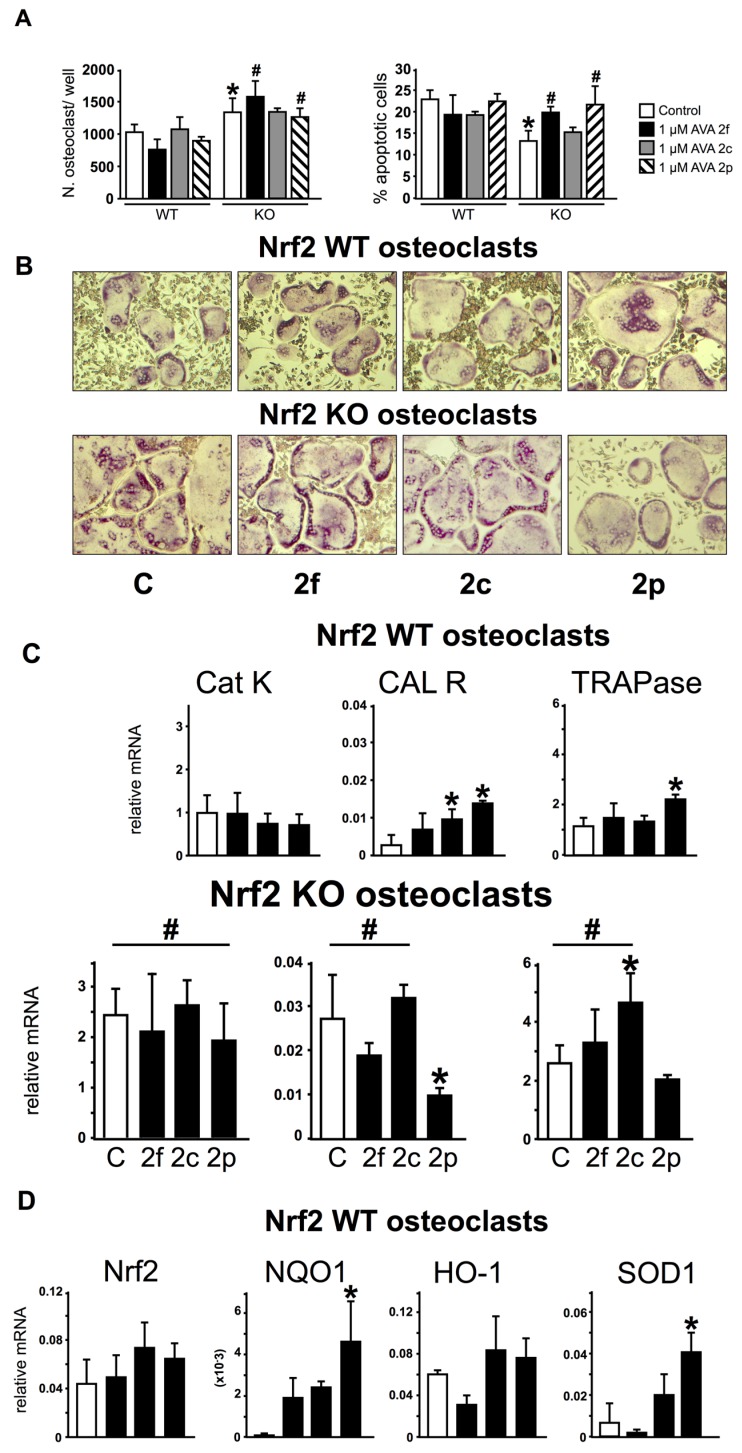

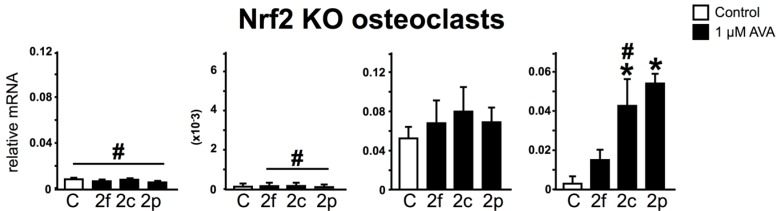
AVAs effect on the regulation of osteoclast gene expression and survival does not require Nrf2 expression. Non adherent cells were differentiated into osteoclasts by treatment with Macrofage Colony Stimulating Factor (M-CSF) and Receptor Activator for Nuclear Factor κB Ligand (sRANKL) for 5 days and treated for 24-h with AVAs. (**A**) Osteoclast number and percentage of dead osteoclasts were quantified by TRAPase-Hematoxylin staining *n* = 3 replicates/treatment; (**B**) Representative images of osteoclasts stained for TRAP are shown; (**C**,**D**) Gene expression in Nrf2 WT and KO cells. mRNA levels were measured by qPCR and corrected by GAPDH. Bars represent means ± SD, *n* = 5 replicates/treatment. * *p* < 0.05 versus cells treated with vehicle by One-Way ANOVA, followed by pairwise multiple comparisons using Holm-Sidak method. ^#^ *p* < 0.05 versus the corresponding WT treatment by Two-Way ANOVA, followed by pairwise multiple comparisons using Student-Newman-Keuls method.

We next examined the effect of AVAs on osteoclasts and the potential requirement of Nrf2. Bone marrow osteoclast precursors lacking Nrf2 (KO cultures) treated with vehicle (control) produced 30% ± 2% more osteoclasts than WT control cultures (Figure 4A,B). In addition, KO control cultures exhibited lower number of apoptotic osteoclasts compared to WT control cultures (13% ± 2% vs. 23% ± 2% for KO and WT, respectively). Although AVAs did not affect the number or the percentage of dead osteoclasts in WT cultures (Figure 4A,B), AVAs 2f and 2p increased the percentage of apoptotic osteoclasts in KO cultures to reach levels found in WT cultures. Consistent with the increased osteoclast number observed in KO cultures, the expression of genes that characterize mature osteoclasts, including Cat K, CAL R and TRAPase, were higher in these preparations compared to WT cultures (Figure 4C). AVA 2f did not affect osteoclastic gene expression either in WT control or KO cultures. AVA 2c increased CAL R expression in WT cultures. Further, AVA 2p increased the expression of CAL R and TRAPase in WT cultures, whereas it decreased CAL R in KO cultures. This latter effect is consistent with the pro-apoptotic effect of AVA 2p on KO cultures (Figure 4A). 

As expected, KO cultures exhibit minimal expression of Nrf2 mRNA compared to WT cultures (Figure 4D). AVAs did not change Nrf2 expression in cultures of either genotype. In addition, NQO1 was the only antioxidant enzyme whose expression was dependent on Nrf2, as mRNA NQO1 transcripts were markedly reduced in KO compared to WT cultures (Figure 4D). In contrast, HO-1 and SOD1 expressions were similar in cultures of both genotypes. AVA 2p increased NQO1 expression in WT, but not in KO cultures. Similar results were obtained with AVA 2f and 2c, although the increase in NQO1 expression did not reach significance in WT cultures. AVAs did not alter HO-1 in cells of either genotype. In contrast, AVAs increased SOD1 expression in both WT and KO cultures, with AVA 2p exhibiting the strongest effect. Taken together, these results show a differential effect of AVAs on the expression of antioxidant enzymes that depends on the enzyme and of the presence or absence of Nrf2.

## 4. Discussion

The results of this study demonstrate that AVAs, compounds found uniquely in oats, regulate the expression of some genes in osteoblastic cells and affect the life span of the bone cells, osteoblasts, osteocytes and osteoclasts. Remarkably, AVAs were similarly effective in WT or Nrf2 KO cells to exert their anti-apoptotic effects on osteoblastic cells and their pro-apoptotic effects on osteoclasts. Although previous studies have shown that AVAs exert cytoprotective effects on other cell types in vitro [50,51], to our knowledge, this is the first study that evaluates the effects of AVAs on bone cell survival.

Osteoblasts express extracellular matrix proteins such as alkaline phosphatase, OCN and COL1A during the cell proliferation, matrix maturation, and mineralization phases [52]. The levels of mRNA expression of these osteoblast markers allows distinguishing the stages of differentiation and maturation of osteoblasts. COL1A among others, is secreted during the early stage of osteogenic differentiation, whereas OCN is a marker of a mature osteoblasts [53,54,55]. Our results revealed that AVA 2c and 2p potentially enhanced osteogenic differentiation by increasing the expression levels of COL1A. Cells of the osteoblastic lineage, osteoblasts, osteocytes and stromal/osteoblastic cells, play an important role in bone remodeling by expressing pro- and anti-osteoclastogenic cytokines [56,57,58,59]. These cells express the master osteoclast differentiation factor RANKL in response to osteoclast-stimulating hormones and cytokines, including PTH, tumor necrosis factor and interleukin-1; and they also express the RANKL decoy receptor OPG that inhibits osteoclastogenesis [45,58,60,61,62]. OPG protects bone from excessive resorption by preventing RANKL from binding to its receptor (RANK) [63]. In our study, the lowest doses of all three AVAs increased OPG in OB-6 cells, suggesting a beneficial effect of AVAs by inhibiting osteoclast differentiation. Furthermore, we found that AVAs 2f and 2c decrease RANKL expression in MLO-Y4 osteocytic cells. Taken together these findings suggest that AVAs could modulate osteoclastogenesis by altering the expression of RANKL and OPG. Future studies are required to examine the relevance of our in vitro findings for the potential beneficial effects of AVAs on the skeleton in vivo; and the mechanistic basis of the differential effects of individual AVAs on bone cell gene expression. 

Apoptosis plays a central role in the maintenance of skeletal mass and strength and several molecular mechanisms are involved in apoptosis regulation in bone cells [64,65]. Changes in the prevalence of osteoblast apoptosis have a significant impact in the number of osteoblasts present at sites of bone formation and their function [66]. Hence, increased osteoblast apoptosis is at least partially responsible for the decreased bone formation associated with the osteopenia induced by glucocorticoid excess [67], and conversely, inhibition of osteoblast apoptosis might contribute to the anabolic effect of intermittent administration of PTH [40,68]. Several studies have emphasized the importance of osteocyte viability for the maintenance of bone structure and strength [69]. Accumulation of apoptotic osteocytes preceded the increase in osteoclasts, suggesting a cause-effect relationship between dead osteocytes and bone resorption [70,71]. Thus, the increased bone fragility that occurs as consequence of glucocorticoid excess, sex steroid deficiency, immobilization and aging, is associated with increased osteoblast and osteocyte apoptosis [72]. It is known that osteoclasts die by apoptosis after completing a bone resorption cycle and that the majority of osteoblasts also die, whereas the remainders become lining cells or osteocytes. Osteocytes also can die prematurely with devastating consequences for bone fragility. Furthermore, it is recognized that systemic hormones, local growth factors, cytokines and pharmacological agents, as well as physical stimuli such as mechanical forces regulate the rate of bone cell apoptosis [66]. The data presented in this report indicate that AVAs do not induce apoptosis of osteoblastic or osteocytic cells. However, AVAs inhibit the effect of several pro-apoptotic stimuli. Moreover, AVA 2p induced apoptosis of KO osteoclasts. Similar to our findings with osteoclasts, AVA 2p induced apoptosis of the human cervical cancer cell line HeLa [73]. Because exaggerated ROS induces apoptosis of osteoblasts and osteocytes whereas it preserves osteoclast viability, it is possible that AVA actions are mediated by their antioxidant properties. 

The Nrf2 pathway constitutes one of the major cellular defense mechanisms against oxidative stress, as evidenced by the fact that mice lacking Nrf2 are prone to the damaging effects of oxidative stress in different tissues [18,74]. The Nrf2 signaling pathway is emerging as a critical factor in the regulation of bone metabolism [75]. Deletion of Nrf2 suppresses antioxidant enzymes and elevates the intracellular ROS level in osteoclasts, increasing osteoclast number and stimulating osteoclast activity [76,77]. Consistent with this previous evidence, we found that bone marrow precursors in KO cultures presented higher number of osteoclasts and that the lack of Nrf2 also enhances osteoclast survival. Furthermore, we showed that AVAs induce osteoclast apoptosis in KO cultures. These findings are consistent with the fact that ROS is required for osteoclast generation and survival. On the other hand, WT, as well as KO primary osteoblastic cells treated with AVAs, were protected from apoptosis. This finding suggests that the pro-survival effect of AVAs on osteoblastic cells as well as the pro-apoptotic effect on osteoclasts does not require Nrf2 expression. Collectively, these outcomes demonstrate that AVAs, at the concentrations and exposure times tested, act by mechanisms independent of Nrf2 in bone cells of both osteoblastic and osteoclastic lineage. Future experiments are needed to determine whether the effective concentrations of AVAs in the current study are found in the bone tissue after dietary intakes of these compounds. 

We also found that NQO1 was the only antioxidant enzyme which expression is strictly dependent on Nrf2; and that the expression of other antioxidant enzymes, including HO-1 and SOD1 was still high in KO cultures, strongly suggesting that they are controlled by alternative factors. AVAs did not have a major effect on the expression of these enzymes, recognized as Nrf2 target genes. However, AVA 2p increased NQO1 in WT cultures and SOD1 in both WT and KO cultures, suggesting that it activates the endogenous antioxidant defense by a mechanism that does not involve Nrf2. 

Our results demonstrating actions of AVA independent of Nrf2 appear to be inconsistent with findings demonstrating that the increase in the expression of the antioxidant enzyme HO-1 induced by AVAs is associated with Nrf2 nuclear translocation in human kidney cells [36]. This difference could be due to the cell type, as well as the dose and time of treatment. 

## 5. Conclusions

In conclusion, although further studies are needed to examine the mechanism(s) by which AVAs regulate bone cell survival, our findings demonstrate that AVAs affect gene expression in bone cells in vitro, as well as cell viability, preventing osteoblastic and osteocytic cell apoptosis and increasing osteoclast apoptosis; and that these effects of AVAs in the studied cells are not mediated by Nrf2.

## References

[1] Lin H., Gao X., Chen G., Sun J., Chu J., Jing K., Li P., Zeng R., Wei B. (2015). Indole-3-carbinol as inhibitors of glucocorticoid-induced apoptosis in osteoblastic cells through blocking ROS-mediated Nrf2 pathway. Biochem. Biophys. Res. Commun..

[2] Benz C.C., Yau C. (2008). Ageing, oxidative stress and cancer: Paradigms in parallax. Nat. Rev. Cancer.

[3] Tarozzi A., Angeloni C., Malaguti M., Morroni F., Hrelia S., Hrelia P. (2013). Sulforaphane as a potential protective phytochemical against neurodegenerative diseases. Oxid. Med. Cell. Longev..

[4] Almeida M. (2012). Aging mechanisms in bone. Bonekey Rep..

[5] Almeida M., Han L., Ambrogini E., Weinstein R.S., Manolagas S.C. (2011). Glucocorticoids and tumor necrosis factor (TNF) alpha increase oxidative stress and suppress WNT signaling in osteoblasts. J. Biol. Chem..

[6] Callaway D.A., Jiang J.X. (2015). Reactive oxygen species and oxidative stress in osteoclastogenesis, skeletal aging and bone diseases. J. Bone Miner. Metab..

[7] Bai X.C., Lu D., Liu A.L., Zhang Z.M., Li X.M., Zou Z.P., Zeng W.S., Cheng B.L., Luo S.Q. (2005). Reactive oxygen species stimulates receptor activator of NF-kappaB ligand expression in osteoblast. J. Biol. Chem..

[8] Lean J.M., Jagger C.J., Kirstein B., Fuller K., Chambers T.J. (2005). Hydrogen peroxide is essential for estrogen-deficiency bone loss and osteoclast formation. Endocrinology.

[9] Xu Z.S., Wang X.Y., Xiao D.M., Hu L.F., Lu M., Wu Z.Y., Bian J.S. (2011). Hydrogen sulfide protects MC3T3-E1 osteoblastic cells against H_2_O_2_-induced oxidative damage-implications for the treatment of osteoporosis. Free Radic. Biol. Med..

[10] Mody N., Parhami F., Sarafian T.A., Demer L.L. (2001). Oxidative stress modulates osteoblastic differentiation of vascular and bone cells. Free Radic. Biol. Med..

[11] Bai X., Lu D., Bai J., Zheng H., Ke Z., Li X., Luo S. (2004). Oxidative stress inhibits osteoblastic differentiation of bone cells by ERK and NF-kappaB. Biochem. Biophys. Res. Commun..

[12] Arai M., Shibata Y., Pugdee K., Abiko Y., Ogata Y. (2007). Effects of reactive oxygen species (ROS) on antioxidant system and osteoblastic differentiation in MC3T3-E1 cells. IUBMB Life.

[13] Almeida M., Han L., Martin-Millan M., Plotkin L.I., Stewart S.A., Roberson P.K., Kousteni S., O’Brien C.A., Bellido T., Parfitt A.M. (2007). Skeletal involution by age-associated oxidative stress and its acceleration by loss of sex steroids. J. Biol. Chem..

[14] Almeida M., Han L., Ambrogini E., Bartell S.M., Manolagas S.C. (2010). Oxidative stress stimulates apoptosis and activates NF-κB in osteoblastic cells via a PKCβ/p66shc signaling cascade: Counter regulation by estrogens or androgens. Mol. Endocrinol..

[15] Mazziotti G., Bilezikian J., Canalis E., Cocchi D., Giustina A. (2012). New understanding and treatments for osteoporosis. Endocrine.

[16] Ibanez L., Ferrandiz M.L., Brines R., Guede D., Cuadrado A., Alcaraz M.J. (2014). Effects of Nrf2 deficiency on bone microarchitecture in an experimental model of osteoporosis. Oxid. Med. Cell. Longev..

[17] Jung K.A., Kwak M.K. (2010). The Nrf2 system as a potential target for the development of indirect antioxidants. Molecules.

[18] Sun Y.X., Xu A.H., Yang Y., Li J. (2015). Role of Nrf2 in bone metabolism. J. Biomed. Sci..

[19] Park C.K., Lee Y., Kim K.H., Lee Z.H., Joo M., Kim H.H. (2014). Nrf2 is a novel regulator of bone acquisition. Bone.

[20] Pandey K.B., Rizvi S.I. (2009). Plant polyphenols as dietary antioxidants in human health and disease. Oxid. Med. Cell. Longev..

[21] Arts I.C., Hollman P.C. (2005). Polyphenols and disease risk in epidemiologic studies. Am. J. Clin. Nutr..

[22] Scalbert A., Holvoet S., Mercenier A. (2005). Dietary polyphenols and the prevention of diseases. Crit. Rev. Food Sci. Nutr..

[23] Pawlowski J.W., Martin B.R., McCabe G.P., Ferruzzi M.G., Weaver C.M. (2014). Plum and soy aglycon extracts superior at increasing bone calcium retention in ovariectomized sprague dawley rats. J. Agric. Food Chem..

[24] Boz H. (2015). Phenolic amides (avenanthramides) in Oats—A review. Czech J. Food Sci..

[25] Meydani M. (2009). Potential health benefits of avenanthramides of oats. Nutr. Rev..

[26] Emmons C.L., Peterson D.M., Paul G.L. (1999). Antioxidant capacity of oat (*Avena sativa* L.) extracts. 2. In vitro antioxidant activity and contents of phenolic and tocol antioxidants. J. Agric. Food Chem..

[27] Mourikis P., Sambasivan R., Castel D., Rocheteau P., Bizzarro V., Tajbakhsh S. (2012). A critical requirement for notch signaling in maintenance of the quiescent skeletal muscle stem cell state. Stem Cells.

[28] Lee-Manion A.M., Price R.K., Strain J.J., Dimberg L.H., Sunnerheim K., Welch R.W. (2009). In vitro antioxidant activity and antigenotoxic effects of avenanthramides and related compounds. J. Agric. Food Chem..

[29] Collins F.W. (1989). Oat phenolics: Avenanthramides, novel substituted N-cinnamoylanthranilate alkaloids from oat groats and hulls. J. Agric. Food Chem..

[30] Bryngelsson S., Dimberg L.H., Kamal-Eldin A. (2002). Effects of commercial processing on levels of antioxidants in oats (*Avena sativa* L.). J. Agric. Food Chem..

[31] Chen C.Y., Milbury P.E., Kwak H.K., Collins F.W., Samuel P., Blumberg J.B. (2004). Avenanthramides and phenolic acids from oats are bioavailable and act synergistically with vitamin C to enhance hamster and human LDL resistance to oxidation. J. Nutr..

[32] Wang P., Chen H., Zhu Y., McBride J., Fu J., Sang S. (2015). Oat avenanthramide-C (2c) is biotransformed by mice and the human microbiota into bioactive metabolites. J. Nutr..

[33] Liu L., Zubik L., Collins F.W., Marko M., Meydani M. (2004). The antiatherogenic potential of oat phenolic compounds. Atherosclerosis.

[34] Koenig R., Dickman J.R., Kang C., Zhang T., Chu Y.F., Ji L.L. (2014). Avenanthramide supplementation attenuates exercise-induced inflammation in postmenopausal women. Nutr. J..

[35] Ren Y., Yang X., Niu X., Liu S., Ren G. (2011). Chemical characterization of the avenanthramide-rich extract from oat and its effect on d-galactose-induced oxidative stress in mice. J. Agric. Food Chem..

[36] Fu J., Zhu Y., Yerke A., Wise M.L., Johnson J., Chu Y., Sang S. (2015). Oat avenanthramides induce heme oxygenase-1 expression via Nrf2-mediated signaling in HK-2 cells. Mol. Nutr. Food Res..

[37] Nie L., Wise M., Peterson D., Meydani M. (2006). Mechanism by which avenanthramide-c, a polyphenol of oats, blocks cell cycle progression in vascular smooth muscle cells. Free Radic. Biol. Med..

[38] Lezcano V., Bellido T., Plotkin L.I., Boland R., Morelli S. (2012). Role of connexin 43 in the mechanism of action of alendronate: Dissociation of anti-apoptotic and proliferative signaling pathways. Arch. Biochem. Biophys..

[39] Lecanda F., Warlow P.M., Sheikh S., Furlan F., Steinberg T.H., Civitelli R. (2000). Connexin43 deficiency causes delayed ossification, craniofacial abnormalities, and osteoblast dysfunction. J. Cell Biol..

[40] Bellido T., Ali A.A., Plotkin L.I., Fu Q., Gubrij I., Roberson P.K., Weinstein R.S., O’Brien C.A., Manolagas S.C., Jilka R.L. (2003). Proteasomal degradation of Runx2 shortens parathyroid hormone-induced anti-apoptotic signaling in osteoblasts. A putative explanation for why intermittent administration is needed for bone anabolism. J. Biol. Chem..

[41] Lecka-Czernik B., Gubrij I., Moerman E.A., Kajkenova O., Lipschitz D.A., Manolagas S.C., Jilka R.L. (1999). Inhibition of Osf2/Cbfa1 expression and terminal osteoblast differentiation by PPAR-gamma 2. J. Cell. Biochem..

[42] Plotkin L.I., Weinstein R.S., Parfitt A.M., Roberson P.K., Manolagas S.C., Bellido T. (1999). Prevention of osteocyte and osteoblast apoptosis by bisphosphonates and calcitonin. J. Clin. Investig..

[43] Kato Y., Windle J.J., Koop B.A., Mundy G.R., Bonewald L.F. (1997). Establishment of an osteocyte-like cell line, MLO-Y4. J. Bone Miner. Res..

[44] Pacheco-Costa R., Hassan I., Reginato R.D., Davis H.M., Bruzzaniti A., Allen M.R., Plotkin L.I. (2014). High bone mass in mice lacking Cx37 due to defective osteoclast differentiation. J. Biol. Chem..

[45] Ben-Awadh A., Delgado-Calle J., Tu X., Kuhlenschmidt K., Allen M.R., Plotkin L.I., Bellido T. (2014). Parathyroid hormone receptor signaling induces bone resorption in the adult skeleton by directly regulating the RANKL gene in osteocytes. Endocrinology.

[46] O’Brien C.A., Plotkin L.I., Galli C., Goellner J., Gortazar A.R., Allen M.R., Robling A.G., Bouxsein M., Schipani E., Turner C.H. (2008). Control of bone mass and remodeling by PTH receptor signaling in osteocytes. PLoS ONE.

[47] Bellido T., Plotkin L.I., Bruzzaniti A., Burr D., Allen M. (2014). Bone cells. Basic and Applied Bone Biology.

[48] Bellido T., Plotkin L.I., Westendorf J.J. (2007). Detection of apoptosis of bone cells in vitro. Osteoporosis.

[49] Plotkin L.I., Mathov I., Aguirre J.I., Parfitt A.M., Manolagas S.C., Bellido T. (2005). Mechanical stimulation prevents osteocyte apoptosis: Requirement of integrins, Src kinases and ERKs. Am. J. Physiol. Cell Physiol..

[50] Guo W., Wise M.L., Collins F.W., Meydani M. (2008). Avenanthramides, polyphenols from oats, inhibit IL-1beta-induced NF-kappaB activation in endothelial cells. Free Radic. Biol. Med..

[51] Lv N., Song M.Y., Lee Y.R., Choi H.N., Kwon K.B., Park J.W., Park B.H. (2009). Dihydroavenanthramide D protects pancreatic beta-cells from cytokine and streptozotocin toxicity. Biochem. Biophys. Res. Commun..

[52] Hu Y., Tang X.X., He H.Y. (2013). Gene expression during induced differentiation of sheep bone marrow mesenchymal stem cells into osteoblasts. Genet. Mol. Res..

[53] Balcerzak M., Hamade E., Zhang L., Pikula S., Azzar G., Radisson J., Bandorowicz-Pikula J., Buchet R. (2003). The roles of annexins and alkaline phosphatase in mineralization process. Acta Biochim. Pol..

[54] Janssens K., Ten D.P., Janssens S., Van H.W. (2005). Transforming growth factor-beta1 to the bone. Endocr. Rev..

[55] Osyczka A.M., Leboy P.S. (2005). Bone morphogenetic protein regulation of early osteoblast genes in human marrow stromal cells is mediated by extracellular signal-regulated kinase and phosphatidylinositol 3-kinase signaling. Endocrinology.

[56] Jimi E., Nakamura I., Amano H., Taguchi Y., Tsurkai T., Tamura M., Takahasi N., Suda T. (1996). Osteoclast function is activated by osteoblastic cells through a mechanism involving cell-to-cell contact. Endocrinology.

[57] Udagawa N., Takahashi N., Jimi E., Matsuzaki K., Tsurukai T., Itoh K., Nakagawa N., Yasuda H., Goto M., Tsuda E. (1999). Osteoblasts/stromal cells stimulate osteoclast activation through expression of osteoclast differentiation factor/RANKL but not macrophage colony-stimulating factor: Receptor activator of NF-kappa B ligand. Bone.

[58] Nakashima T., Hayashi M., Fukunaga T., Kurata K., Oh-hora M., Feng J.Q., Bonewald L.F., Kodama T., Wutz A., Wagner E.F. (2011). Evidence for osteocyte regulation of bone homeostasis through RANKL expression. Nat. Med..

[59] Xiong J., Onal M., Jilka R.L., Weinstein R.S., Manolagas S.C., O’Brien C.A. (2011). Matrix-embedded cells control osteoclast formation. Nat. Med..

[60] Simonet W.S., Lacey D.L., Dunstan C.R., Kelley M., Chang M.S., Luthy R., Nguyen H.Q., Wooden S., Bennett L., Boone T. (1997). Osteoprotegerin: A novel secreted protein involved in the regulation of bone density. Cell.

[61] Kramer I., Halleux C., Keller H., Pegurri M., Gooi J.H., Weber P.B., Feng J.Q., Bonewald L.F., Kneissel M. (2010). Osteocyte Wnt/beta-catenin signaling is required for normal bone homeostasis. Mol. Cell Biol..

[62] Zhao S., Zhang Y.K., Harris S., Ahuja S.S., Bonewald L.F. (2002). MLO-Y4 osteocyte-like cells support osteoclast formation and activation. J. Bone Miner. Res..

[63] Theoleyre S., Wittrant Y., Tat S.K., Fortun Y., Redini F., Heymann D. (2004). The molecular triad OPG/RANK/RANKL: Involvement in the orchestration of pathophysiological bone remodeling. Cytokine Growth Factor Rev..

[64] Jilka R.L., Bellido T., Almeida M., Plotkin L.I., O’Brien C.A., Weinstein R.S., Manolagas S.C., Bilezikian J.P., Raisz L.G., Martin T.J. (2008). Apoptosis in bone cells. Principles of Bone Biology.

[65] Bilezikian J.P., Raisz L.G., Rodan G.A. (1996). Principles of Bone Biology.

[66] Bellido T., Plotkin L.I. (2011). Novel actions of bisphosphonates in bone: Preservation of osteoblast and osteocyte viability. Bone.

[67] Weinstein R.S., Jilka R.L., Parfitt A.M., Manolagas S.C. (1998). Inhibition of osteoblastogenesis and promotion of apoptosis of osteoblasts and osteocytes by glucocorticoids: Potential mechanisms of their deleterious effects on bone. J. Clin. Investig..

[68] Jilka R.L., Weinstein R.S., Bellido T., Roberson P., Parfitt A.M., Manolagas S.C. (1999). Increased bone formation by prevention of osteoblast apoptosis with parathyroid hormone. J. Clin. Investig..

[69] Plotkin L.I. (2014). Apoptotic osteocytes and the control of targeted bone resorption. Curr. Osteoporos. Rep..

[70] Aguirre J.I., Plotkin L.I., Stewart S.A., Weinstein R.S., Parfitt A.M., Manolagas S.C., Bellido T. (2006). Osteocyte apoptosis is induced by weightlessness in mice and precedes osteoclast recruitment and bone loss. J. Bone Miner. Res..

[71] Verborgt O., Gibson G., Schaffler M.B. (2000). Loss of osteocyte integrity in association with microdamage and bone remodeling after fatigue in vivo. J. Bone Miner. Res..

[72] Plotkin L.I., Bivi N., Bellido T. (2011). A bisphosphonate that does not affect osteoclasts prevents osteoblast and osteocyte apoptosis and the loss of bone strength induced by glucocorticoids in mice. Bone.

[73] Wang D., Wise M.L., Li F., Dey M. (2012). Phytochemicals attenuating aberrant activation of beta-catenin in cancer cells. PLoS ONE.

[74] Motohashi H., Yamamoto M. (2004). Nrf2-Keap1 defines a physiologically important stress response mechanism. Trends Mol. Med..

[75] Sun Y.X., Li L., Corry K.A., Zhang P., Yang Y., Himes E., Mihuti C.L., Nelson C., Dai G., Li J. (2015). Deletion of Nrf2 reduces skeletal mechanical properties and decreases load-driven bone formation. Bone.

[76] Hyeon S., Lee H., Yang Y., Jeong W. (2013). Nrf2 deficiency induces oxidative stress and promotes RANKL-induced osteoclast differentiation. Free Radic. Biol. Med..

[77] Kanzaki H., Shinohara F., Kajiya M., Kodama T. (2013). The Keap1/Nrf2 protein axis plays a role in osteoclast differentiation by regulating intracellular reactive oxygen species signaling. J. Biol. Chem..

